# Supplementary material for: Outcomes Associated with Respiratory Failure for Patients with Cardiogenic Shock and Acute Myocardial Infarction: A Substudy of the CULPRIT-SHOCK Trial
Source: J Clin Med. 2020 Mar 20;9(3):860. doi: 10.3390/jcm9030860 (PMC7141492; doi:10.3390/jcm9030860)
Supplement: Supplementary file 1 [file jcm-09-00860-s001.pdf]

SUPPLEMENTAL MATERIAL:

Table S1. Clinical outcomes at 365 days.

	**No Ventilation n=119**	**Ventilation at admission n=408**	**Ventilation within day 1 after admission n=156**	**p-value**
**Primary endpoint /N (%)**				
Death	32/119 (26.9)	227/407 (55.8)	105/156 (67.3)	<0.001
**Secondary endpoints n/N (%)**				
Renal replacement therapy	9/119 (7.6)	63/407 (15.5)	24/156 (15.4)	0.08
Myocardial Infarction	1/119 (0.8)	11/407 (2.7)	1/156 (0.6)	0.18
Rehospitalization	5/119 (4.2)	12/407 (2.9)	5/156 (3.2)	0.79
Repeat revascularization	35/119 (29.4)	79/407 (19.4)	29/156 (18.6)	0.044
**Safety endpoints n/N (%)**				
Bleeding event	22/119 (18.5)	104/407 (25.6)	35/156 (22.4)	0.26
Stroke	5/119 (4.2)	14/407 (3.4)	10/156 (6.4)	0.29

Table S2. Independent predictors for ventilation.

	Odds Ratio (95% CI)	p-value
**Ventilation at presentation**		
Weight	1.04 (1.01-1.06)	0.003
Resuscitation within 24h	48.51 (21.13-111.35)	<0.001
Left bundle branch block	3.00 (1.01-8.97)	0.048
ST-segment elevation	0.43 (0.21-0.90)	0.024
Heart rate	1.02 (1.00-1.03)	0.012
Catecholamine requirement	23.90 (8.94-63.91)	<0.001
**Ventilation within day 1 after admission**		
Heart rate	1.01 (1.00-1.02)	0.009
Catecholamine requirement	9.36 (4.09-21.43)	<0.001

CI = confidence interval.

Table S3. Characteristics of non-resuscitated patients.

	No Ventilation n =101	Ventilation at admission n=85	Ventilation within day 1 after admission n=129	p-value
**Age – years**				
Median (IQR)	70 (62,77)	71 (63,78)	74 (64,80)	0.09
**BMI kg/m²**				
Median (IQR)	25.6 (22.9,28.6)	27.8 (25.4,31.1)	26.7 (24.6,29.4)	0.003
**Cardiovascular risk factors n/N (%)**				
Current smoking	37/100 (37.0)	21/82 (25.6)	26/123 (21.1)	0.028
Hypertension	59/101 (58.4)	55/83 (66.3)	81/125 (64.8)	0.48
Dyslipidemia	36/101 (35.6)	33/82 (40.2)	37/125 (29.6)	0.27
Diabetes mellitus	32/101 (31.7)	34/82 (41.5)	47/126 (37.3)	0.38
Family history of CAD	62/100 (16.0)	6/81 (7.4)	10/120 (8.3)	0.10
**Previous myocardial infarction n/N (%)**	21/101 (20.8)	17/82 (20.7)	19/127 (15.0)	0.43
**Previous stroke n/N (%)**	6/101 (5.9)	9/82 (11.0)	12/128 (9.4)	0.45
**Known peripheral artery disease n/N (%)**	14/101 (13.9)	15/83 (18.1)	20/128 (15.6)	0.74
**Known renal insufficiency (GFR < 30ml/min) n/N (%)**	6/101 (5.9)	9/83 (10.8)	11/126 (8.7)	0.48
**Chronic dialysis n/N (%)**	1/101 (1.0)	1/83 (1.2)	2/128 (1.6)	0.93
**Previous PCI n/N (%)**	21/101 (20.8)	24/82 (29.3)	16/127 (12.6)	0.012
**Previous CABG no (%) n/N (%)**	3/101 (3.0)	5/83 (6.0)	2/127 (1.6)	0.20
**Signs of impaired organ perfusion n/N (%)**				
Altered mental status	41/101 (40.6)	49/84 (58.3)	55/129 (42.6)	0.032
Cold, clammy skin and limbs	54/101 (53.5)	51/82 (62.2)	83/128 (64.8)	0.20
Oliguria (≤ 30ml/h)	9/100 (9.0)	29/80 (36.3)	33/123 (26.8)	<0.001
Arterial lactate >2.0 mmol/l	42/100 (42.0)	57/81 (70.4)	90/125 (72.0)	<0.001
**ST-segment elevation n/N (%)**	73/99 (73.7)	41/83 (49.4)	89/128 (69.5)	0.001
anterior **n/N (%)**	32/72 (44.4)	23/41 (56.1)	51/88 (58.0)	0.21
non-anterior **n/N (%)**	40/72 (55.6)	18/41 (43.9)	37/88 (42.0)	0.21
**ST-segment depression n/N (%)**	39/99 (39.4)	46/83 (55.4)	63/128 (49.2)	0.089
**LBBB n/N (%)**	9/99 (9.1)	16/83 (19.3)	14/128 (10.9)	0.91
**Mean arterial pressure – mmHg**				

Median (IQR)	78 (63, 93)	73 (62,88)	75 (62, 93)	0.57
**Heart rate-beats/min**				
Median (IQR)	80 (60, 107)	91 (79, 107)	95 (77, 109)	0.005
**Creatinine (umol/l)**				
Median (IQR)	98.0 (80.0, 129.6)	119.7 (96.6, 157.2)	120.7 (92.0, 163.0)	0.033
**N° affected vessels n/N (%)**				0.004
1 **n/N (%)**	2/101 (2.0)	1/85 (1.2)	0/129 (0.0)	
2 **n/N (%)**	47/101 (46.5)	20/85 (23.5)	51/129 (39.5)	
3 **n/N (%)**	52/101 (51.5)	64/85 (75.3)	78/129 (60.5)	
**Artery with culprit lesion n/N (%)**				0.35
Left anterior descending	34/101 (33.7)	30/85 (35.3)	55/129 (42.6)	
Left circumflex	18/101 (17.8)	16/85 (18.8)	21/129 (16.3)	
Right coronary	72/101 (41.6)	26/85 (30.6)	37/28.7 (28.7)	
Left main	7/101 (6.9)	10/85 (11.8)	16/129 (12.4)	
Bypass	0/101 (0.0)	3/85 (3.5)	0/129 (0.0)	
**Left ventricular ejection fraction-%**				
Median (IQR)	39 (30, 50)	32 (20, 40)	30 (20, 37)	0.014
**Primary endpoint of death at 30 days n/N (%)**	20/101 (19.8)	44/85 (51.8)	83/129 (64.3)	<0.001

IQR= interquartile range; BMI=body mass index; CAD=coronary artery disease GFR=glomerular filtration rate; PCI=percutaneous coronary intervention; CABG=coronary artery bypass graft; LBBB=left bundle branch block.

Table S4. Baseline characteristics according to ventilation.

	Never ventilated	NIV alone	NIV before IMV	IMV alone
	n=119	n=56	n=12	n=435
**Age – years**				
Median (IQR)	70 (60, 78)	68 (58,76)	77 (69,84)	70 (61,78)
**BMI kg/m2**				
Median (IQR)	25.7 (23.4,28.4)	26.0 (23.5,28.7)	26.5 (25.9,30.3)	27.1 (24.7,29.4)
**Cardiovascular risk factors n/N (%)**				
Current smoking	43/118 (36.4)	16/51 (31.4)	3/12 (25.0)	98/418 (23.4)
Hypertension	69/169 (58.0)	36/54 (66.7)	8/12 (66.7)	255/426 (59.9)
Dyslipidemia	44/119 (37.0)	18/54 (33.3)	3/12 (25.0)	130/423 (30.7)
Diabetes mellitus	38/119 (31.9)	15/54 (27.8)	8/12 (66.7)	139/424 (32.8)
Family history of CAD	19/118 (16.1)	10/48 (20.8)	3/12 (25.0)	39/412 (9.5)
**Previous myocardial infarction n/N (%)**	24/118 (20.3)	7/54 (13.0)	2/12 (16.7)	66/426 (15.5)
**Previous stroke n/N (%)**	9/119 (7.6)	5/54 (9.3)	0/12 (0.0)	31/429 (7.2)
**Known peripheral artery disease n/N (%)**	15/119 (12.6)	6/54 (11.1)	3/12 (25.0)	46/429 (10.7)
**Known renal insufficiency (GFR <30 ml/min) n/N (%)**	6/119 (5.0)	3/54 (5.6)	1/12 (8.3)	32/427 (7.5)
**Chronic dialysis n/N (%)**	1/119 (0.8)	0/55 (0.0)	0/12 (0.0)	4/428 (0.9)
**Previous PCI n/N (%)**	25/118 (21.2)	12/54 (22.2)	1/12 (8.3)	73/426 (17.1)
**Previous CABG no (%) n/N (%)**	4/119 (3.4)	2/55 (3.6)	0/12 (0.0)	22/428 (5.1)
**Signs of impaired organ perfusion n/N (%)**				
Altered mental status	54/119 (45.4)	26/55 (47.3)	9/12 (75.0)	328/434 (75.6)
Cold, clammy skin and limbs	67/119 (56.3)	34/52 (65.4)	10/11 (90.9)	318/433 (73.4)
Oliguria (≤30ml/h)	12/117 (10.3)	15/50 (30.0)	5/11 (45.5)	126/425 (29.6)
Arterial lactate >2.0 mmol/l	49/117 (41.9)	31/51 (60.8)	12/12 (100.0)	303/422 (71.8)
**Resuscitation before randomization n/N (%)**	18/119 (15.1)	16/56 (28.6)	1/12 (8.3)	286/433 (66.1)
**ST-segment elevation n/N (%)**	85/117 (72.6)	38/54 (70.4)	7/10 (70.0)	252/424 (59.4)
anterior **n/N (%)**	40/84 (47.6)	22/38 (57.9)	7/7 (100.0)	132/249 (53.0)
non-anterior **n/N (%)**	44/84 (52.4)	16/38 (42.1)	0/7 (0.0)	117/249 (47.0)
**ST-segment depression n/N (%)**	45/117 (38.5)	25/54 (46.3)	6/10 (60.0)	204/424 (48.1)
**LBBB n/N (%)**	11/117 (9.4)	5/54 (9.3)	1/10 (10.0)	69/425 (16.2)
**Mean arterial pressure – mmHg**				
Median (IQR)	78 (63,93)	80 (67,104)	81 (63,87)	73 (63,91)

**Heart rate - beats/min**				
Median (IQR)	79 (61,101)	96 (68,112)	106 (82,123)	92 (78, 107)
**Creatinine (umol/l)**				
Median (IQR)	97.0 (77.0, 124,6)	106.1 (90.0, 128.2)	155.6 (97.0, 330.2)	118.2 (95.7, 150.3)
**N° affected vessels n/N (%)**				
1 **n/N (%)**	2/119 (1.7)	0/56 (0.0)	0/12 (0.0)	3/434 (0.7)
2 **n/N (%)**	54/119 (45.4)	19/56 (33.9)	6/12 (50.0)	285/434 (65.7)
3 **n/N (%)**	63/119 (52.9)	37/56 (66.1)		
**Artery with culprit lesion n/N (%)**				
Left anterior descending	104/119 (87.4)	47/56 (83.9)	12/12 (100.0)	374/435 (86.0)
Left circumflex	83/119 (69.7)	45/56 (80.4)	7/12 (58.3)	331/435 (76.1)
Right coronary	87/119 (73.1)	44/56 (78.6)	10/12 (83.3)	338/435 (77.7)
Left main	15/119 (12.6)	8/56 (14.3)	3/12 (25.0)	74/435 (17.0)
Bypass	1/119 (0.8)	2/56 (3.6)	0/12 (0.0)	10/435 (2.3)
**Left ventricular ejection fraction - %**				
Median (IQR)	38 (30,48)	30 (21,40)	35 (26,40)	30 (25, 40)
**Primary endpoint of death at 30 days n/N (%)**	25/119 (21.0)	16/55 (29.1)	8/12 (66.7)	263/435 (60.5)

IQR= interquartile range; BMI=body mass index; CAD=coronary artery disease GFR=glomerular filtration rate; PCI=percutaneous coronary intervention; CABG=coronary artery bypass graft; LBBB=left bundle branch block.
